# Supplementary material for: Complexome profiling of the Chlamydomonas psb28 mutant reveals TEF5 as an early PSII assembly factor
Source: Plant Cell. 2025 Mar 17;37(6):koaf055. doi: 10.1093/plcell/koaf055 (PMC12164590; doi:10.1093/plcell/koaf055)
Supplement: koaf055_Supplementary_Data [file koaf055_supplementary_data.zip › psb28_interactive.zip!/plots/intensity/qualityPlots/peptides/HSP70G_Cre10.g439900.t1.2.html]

### Cre10.g439900.t1.2

Trivial name: HSP70G  
SeqCoverage [median]: 9.50  
Mass: 120.89 kDa  
  
 

| Sequence | MissedCleavage | Mass [Da] | wtIntensity | muIntensity | Score |
| --- | --- | --- | --- | --- | --- |
| AKLPDVDVLQLPK | 1 | 1434.84 | 0.00 | 3159.26 | 34.39 |
| EALETDELMQK | 0 | 1305.61 | 0.00 | 350.70 | 62.82 |
| FSVYTVK | 0 | 842.45 | 0.00 | 1043.91 | 73.98 |
| HFAAQFSQK | 0 | 1062.52 | 380.70 | 661.66 | 80.44 |
| LDGVAADPLR | 0 | 1025.55 | 0.00 | 808.65 | 89.05 |
| LQAALSEVLGGR | 0 | 1212.68 | 0.00 | 2409.30 | 86.62 |
| QLAEVEADLNAKEESQSK | 1 | 1987.97 | 0.00 | 498.49 | 34.72 |
| TPISIAVNEMSK | 0 | 1288.67 | 0.00 | 659.95 | 102.06 |
| VSTEEQR | 0 | 847.40 | 259.93 | 0.00 | 94.41 |
| VYNQLEVR | 0 | 1019.54 | 437.51 | 1479.07 | 98.30 |
|  |  |  | 1078.1 | 11071.0 |  |
